# Use of electronic patient records and encrypted email patient communication among Swiss chiropractors: a population-based cross-sectional study

**DOI:** 10.1186/s12998-023-00495-z

**Published:** 2023-07-17

**Authors:** Cesar A. Hincapié, Léonie Hofstetter, Rahim Lalji, Longin Korner, Mireille C. Schläppi, Serafin Leemann

**Affiliations:** 1 EBPI-UWZH Musculoskeletal Epidemiology Research, Balgrist University Hospital and University of Zurich, Zurich, Switzerland; 2 Epidemiology, Biostatistics and Prevention Institute (EBPI), University of Zurich, Zurich, Switzerland; 3 University Spine Centre Zurich (UWZH), Balgrist University Hospital and University of Zurich, Zurich, Switzerland; 4 Swiss Chiropractic Association (ChiroSuisse), Bern, Switzerland; 5 Epidemiology, Biostatistics and Prevention Institute (EBPI) and University Spine Centre Zurich (UWZH), University of Zurich and Balgrist University Hospital, Forchstrasse 340, Zurich, 8008 Switzerland

**Keywords:** Electronic health records, Medical informatics, Health services research, Chiropractic, Healthcare digitalisation

## Abstract

**Background:**

The implementation of electronic health information technologies is a key target for healthcare quality improvement. Among Swiss chiropractors, reliable data on the use of electronic heath information technologies and distribution of the health workforce was lacking.

**Objectives:**

To estimate the prevalence of electronic patient record (EPR) and encrypted email communication use among Swiss chiropractors and describe the geographic distribution of chiropractors in Switzerland.

**Methods:**

Population-based cross-sectional study of all active practising members of the Swiss Chiropractic Association (ChiroSuisse) between 3 December 2019 and 31 January 2020. We asked about clinician and practice characteristics, EPR use for clinical record keeping, use of encrypted email for patient communication, and information on EPR and encrypted email communication products used. Multivariable logistic regression analyses assessed the associations between clinician and practice characteristics and (1) EPR use, and (2) encrypted email use.

**Results:**

Among 286 eligible Swiss chiropractors (193 [68%] men; mean age, 51.4 [SD, 11.2] years), 217 (76%) completed the survey (140 [65%] men; mean age 50.7 [11.2] years). Among respondents, 47% (95% confidence interval [CI], 40–54%) reported using an EPR in their practice, while 60% (95% CI, 54–67%) endorsed using encrypted email technology. Chiropractors aged ≥ 60 (versus those ≤ 39) years were 74% less likely to use an EPR system (OR 0.26, 95% CI 0.08 to 0.77), while clinicians from practices with 4 or more chiropractors (versus those from solo practices) were over 5 times more likely to report EPR use (OR 5.6, 2.1 to 16.5). Findings for factors associated with encrypted email use were similar. The density of chiropractors in Switzerland was 3.3 per 100,000 inhabitants.

**Conclusions:**

As of January 2020, 286 duly licensed chiropractors were available to provide musculoskeletal healthcare in Switzerland — just under 50% of responding Swiss chiropractors used an EPR system in clinical practice, while 60% used encrypted email technology. Better implementation of EPR and electronic health information technologies in Swiss chiropractic practice is possible and encouraged for the purpose of musculoskeletal healthcare quality improvement.

**Supplementary Information:**

The online version contains supplementary material available at 10.1186/s12998-023-00495-z.

## Introduction

Little is known about the use of health digitalisation technologies within the Swiss chiropractic health workforce. The implementation of electronic heath information technologies is seen both within Europe and globally as a key target for healthcare quality improvement [1, 2]. Since 2017, Swiss federal law has required hospitals to adopt an interoperable electronic patient record (EPR) compatible with national standards. Other primary care healthcare providers, such as physicians and chiropractors, have been encouraged to voluntarily contribute to this development [3], and this healthcare digitalisation trend is underway in Switzerland [2, 4] as in most parts of the world. This ongoing paradigm shift toward digitalised healthcare in Switzerland is further confirmed by the recently launched Health2030—the Federal Council’s health policy strategy 2020–2030 [5]. There are many challenges to implementing electronic heath information technologies in clinical practices, such as high initial costs, lack of digital literacy, burden on clinician time and workload, and data privacy and security concerns [6]. Compared to other countries, Switzerland’s adoption of EPR systems has been slow [2], with only 23% of primary care physicians reporting use of an EPR in their practice in 2010 [4].

Musculoskeletal pain conditions, such as low back pain and neck pain, are a leading cause of disability globally and the most common disorders treated by chiropractors in Switzerland [7] and worldwide [8]. One factor which may contribute to the disability burden is the existence of low-value musculoskeletal healthcare — health services that confer little or no benefit to patients or where risk of harm exceeds probable benefit [9]. As a large proportion of musculoskeletal pain is managed in primary care, efforts to improve the quality of care in these settings, such as better uptake and implementation of EPRs and healthcare digitalization technologies, may play an important role in musculoskeletal healthcare quality improvement [10, 11]. Among Swiss chiropractors, reliable data on the use of electronic heath information technologies and distribution of the health workforce was lacking. Our primary objectives were to determine the prevalence of EPR and encrypted email communication use among Swiss chiropractors and examine factors associated with use of these electronic health information technologies. A secondary aim was to describe the geographic distribution of chiropractors in Switzerland.

## Methods

### Study design

We conducted a population-based cross-sectional electronic survey between 3 December 2019 and 31 January 2020, to describe the use of EPRs and encrypted email communication among Swiss chiropractors, as well as the geographic distribution of chiropractors in Switzerland. Our study is reported according to the Strengthening the Reporting of Observational Studies in Epidemiology (STROBE) Statement for cross-sectional studies [12]. The independent research ethics committee of Canton Zurich confirmed that ethical approval was not required for this survey of Swiss chiropractic clinicians pursuant to Art. 2 (outside scope) of the Swiss Federal Act on Research involving Human Beings (Human Research Act, HRA). All methods followed relevant guidelines and regulations.

### Source population

Eligible participants were active full members of the Swiss Chiropractic Association (ChiroSuisse) as indicated by the ChiroSuisse active full members roster list (used with permission from ChiroSuisse) as of 1 December 2019 [13]. Approximately 98% of all chiropractors in Switzerland are members of ChiroSuisse (personal communication, 1 September 2022). Information on clinical experience was based on years since chiropractic license was issued, which was extracted from the publicly available register of medical professions in Switzerland [13]. Language of the clinician was derived from the cantons’ primary language spoken.

### Data collection

An initial survey invitation was sent via email on 3 December 2019, with 4 email reminders sent between 10 December and 27 January 2020. We used the Research Electronic Data Capture (REDCap) [14] web application platform to collect, encrypt, and store all survey data. The surveys were distributed in English, as all Swiss chiropractors are able to communicate in English. In the survey, we asked about clinician and practice characteristics, EPR use for clinical record keeping, use of encrypted email for patient communication, and information on EPR and encrypted email communication products used. Chiropractors’ information about EPR use in their practice was categorised as: (1) complete EPR use; (2) partial EPR use; (3) no EPR use but considering EPR use in the near future; and (4) no EPR use.

### Statistical analysis

We summarised source and study population characteristics using descriptive statistics and reported on geographic distribution at three levels (country, 7 regions, 26 cantons). Categorical data were analysed with counts and proportions with 95% confidence intervals (CI) and continuous data as means and standard deviations (SDs), or medians and interquartile ranges (IQRs), as appropriate. We used multivariable logistic regression analyses to assess the association between clinician and practice characteristics and (1) EPR use and (2) encrypted email use by estimating odds ratios (ORs) and 95% CIs. We also examined characteristics associated with survey participation versus nonparticipation. Age, sex, language, region, and number of chiropractors in practice were used as predictor variables. To create the binary EPR use variable for logistic regression analyses, we recoded the levels “complete EPR use” and “partial EPR use” as “EPR use”, and “considering EPR use” and “no EPR use” as “no EPR use”. All analyses were performed using R version 3.6.1 (R Foundation for Statistical Computing) within an RStudio environment [15]. The choropleth map was generated using the RSwissMaps_0.1.4 package.

## Results

### Characteristics of source and study populations

The eligible source population consisted of 286 Swiss chiropractors (68% men; mean age, 51 ± 11 years), representing 208 unique practices. Our study population comprised 217 chiropractors that completed the survey (76% participation). Characteristics of the source population, survey participants, and nonparticipants are detailed in Table 1. Our study population was quite similar to our source population with respect to age, gender, language, and clinical experience. Compared with participants, nonparticipants were slightly older and more commonly men.

**Table 1:** Characteristics of Swiss chiropractors, study participants, and nonparticipants

Characteristic	Source population (n = 286)		Participants (n = 217)		Nonparticipants (n = 69)	
	N	%	N	%	N	%
Sex						
Female	93	32.5	77	35.5	16	23.2
Male	193	67.5	140	64.5	53	76.8
Age – mean ± SD (y)	51.4 ± 11.2		50.7 ± 11.2		53.9 ± 11.1	
Age groups (y)						
≤ 29	4	1.4	4	1.8	0	0
30–39	34	11.9	27	12.4	7	10.1
40–49	85	29.7	68	31.3	17	24.6
50–59	89	31.1	71	32.7	18	26.1
60–69	57	19.9	35	16.1	22	31.9
≥ 70	14	4.9	10	4.6	4	5.8
NA	3	1.0	2	0.9	1	1.4
Language						
German	185	64.7	140	64.5	45	65.2
French	89	31.1	67	30.9	22	31.9
Italian	12	4.2	10	4.6	2	2.9
Clinical experience – mean ± SD (y)	22.5 ± 10.3		22.1 ± 10.1		23.7 ± 10.8	
Clinical experience groups (y)						
≤ 10	38	13.3	28	12.9	10	14.5
11–20	85	29.7	71	32.7	14	20.3
21–30	109	38.1	79	36.4	30	43.5
31–40	39	13.6	28	12.9	11	15.9
41–50	12	4.2	9	4.1	3	4.3
≥ 50	2	0.7	1	0.5	1	1.4
NA	1	0.3	1	0.5	0	0
Practice size – N chiropractors						
1	NA	NA	102	47.0	NA	NA
2–3	NA	NA	81	37.3	NA	NA
≥ 4	NA	NA	34	15.7	NA	NA

### Prevalence of EPR use and factors associated with EPR use

Eighty participants (36.9%; 95% CI, 30.7 to 43.5%) of 217 survey respondents reported only using an EPR system in their practice, while 22 participants (10.1%, 6.8 to 14.9%) reported mixed use of an EPR and paper-based system. In total, EPR use was reported by 47.0% (40.5 to 53.6%) of study participants. Among 115 chiropractors (53%, 46.4 to 59.5%) that reported using a paper-based system only, 37 (17.1%, 12.6 to 22.6%) endorsed that they were considering switching to an EPR system in the near future. Characteristics of the study population, EPR users, and EPR non-users are detailed in Table 2. The EPR products and manufacturers used are presented in Supplementary Appendix eTable 1. A summary of features (appointment scheduling, imaging and lab interfacing, alert systems, billing, statistics/reporting, patient access portal) for the 5 most commonly used EPR products by Swiss chiropractors is provided in Supplementary Appendix eTable 2. A large majority (91%) of survey respondents also provided information on their billing and invoicing systems. In most cases, the same system used for billing and invoicing was also used for patient record keeping. Further information on billing systems is provided in Supplementary Appendix eTable 3.

Our logistic regression analysis found that chiropractors aged ≥ 60 years were 74% less likely to use an EPR system (OR 0.26; 95% CI, 0.08 to 0.77) compared with those aged ≤ 39 years. Compared with clinicians in solo practices, clinicians from practices with four or more chiropractors were 5.6 times more likely to report EPR use (OR 5.6, 2.1 to 16.5). Our analysis was compatible with no associations between EPR use and sex, language, and region (see Supplementary Appendix eTable 4 for details).

**Table 2:** Characteristics of Swiss chiropractor participants, EPR users, and EPR non-users

Characteristic	Study population (n = 217)		EPR users (n = 102)		EPR non-users (n = 115)	
	N	%	N	%	N	%
Sex						
Female	77	35.5	43	42.2	34	29.6
Male	140	64.5	59	57.8	81	70.4
Age – mean ± SD (y)	50.7 ± 11.2		47.2 ± 10.9		53.8 ± 10.4	
Age (y)						
≤ 29	4	1.8	3	2.9	1	0.9
30–39	27	12.4	18	17.6	9	7.8
40–49	68	31.3	42	41.2	26	22.6
50–59	71	32.7	26	25.5	45	39.1
60–69	35	16.1	9	8.8	26	22.6
≥ 70	10	4.6	4	3.9	6	5.2
NA	2	0.9	0	0	2	1.7
Language						
German	140	64.5	68	66.7	72	62.6
French	67	30.9	28	27.5	39	33.9
Italian	10	4.6	6	5.9	4	3.5
Clinical experience – mean ± SD (y)	22.1 ± 10.1		18.9 ± 10.2		25.1 ± 9.2	
Clinical experience (y)						
≤ 10	28	12.9	22	21.6	6	5.2
11–20	71	32.7	37	36.3	34	29.6
21–30	79	36.4	31	30.4	48	41.7
31–40	28	12.9	9	8.8	19	16.5
41–50	9	4.1	3	2.9	6	5.2
≥ 50	1	0.5	0	0	1	0.9
NA	1	0.5	0	0	1	0.9
Practice size – N chiropractors						
1	102	47.0	39	38.2	63	54.8
2–3	81	37.3	36	35.3	45	30.7
≥ 4	34	15.7	27	26.5	7	6.1

### Prevalence of encrypted email use and factors associated with use

Encrypted email technology use was reported by 60% (95% CI, 53.7 to 66.6%) of participants. Among secure email users, 86% were German-speaking, while 69% of secure email non-users were French- or Italian-speaking (see Supplementary Appendix eTable 5). Health Info Net (HIN) email was the most commonly used secure email communication technology product, with 94% of Swiss chiropractors reporting its use.

Chiropractors aged ≥ 60 years were 75% less likely to report encrypted email use (OR 0.25, 95% CI 0.07 to 0.86) compared with those aged ≤ 39 years. Clinicians from practices with four or more chiropractors were 7.4 times more likely to report encrypted email use (OR 7.4, 2.0 to 33.0) compared with those from solo practices. Italian or French speaking chiropractors were 93% less likely to use encrypted email (OR 0.07, 0.03 to 0.15) compared with German-speaking chiropractors. Our analysis was compatible with no associations between email use and sex (see Supplementary Appendix eTable 6 for details).

### Geographic distribution of swiss chiropractors

**Fig. 1:**
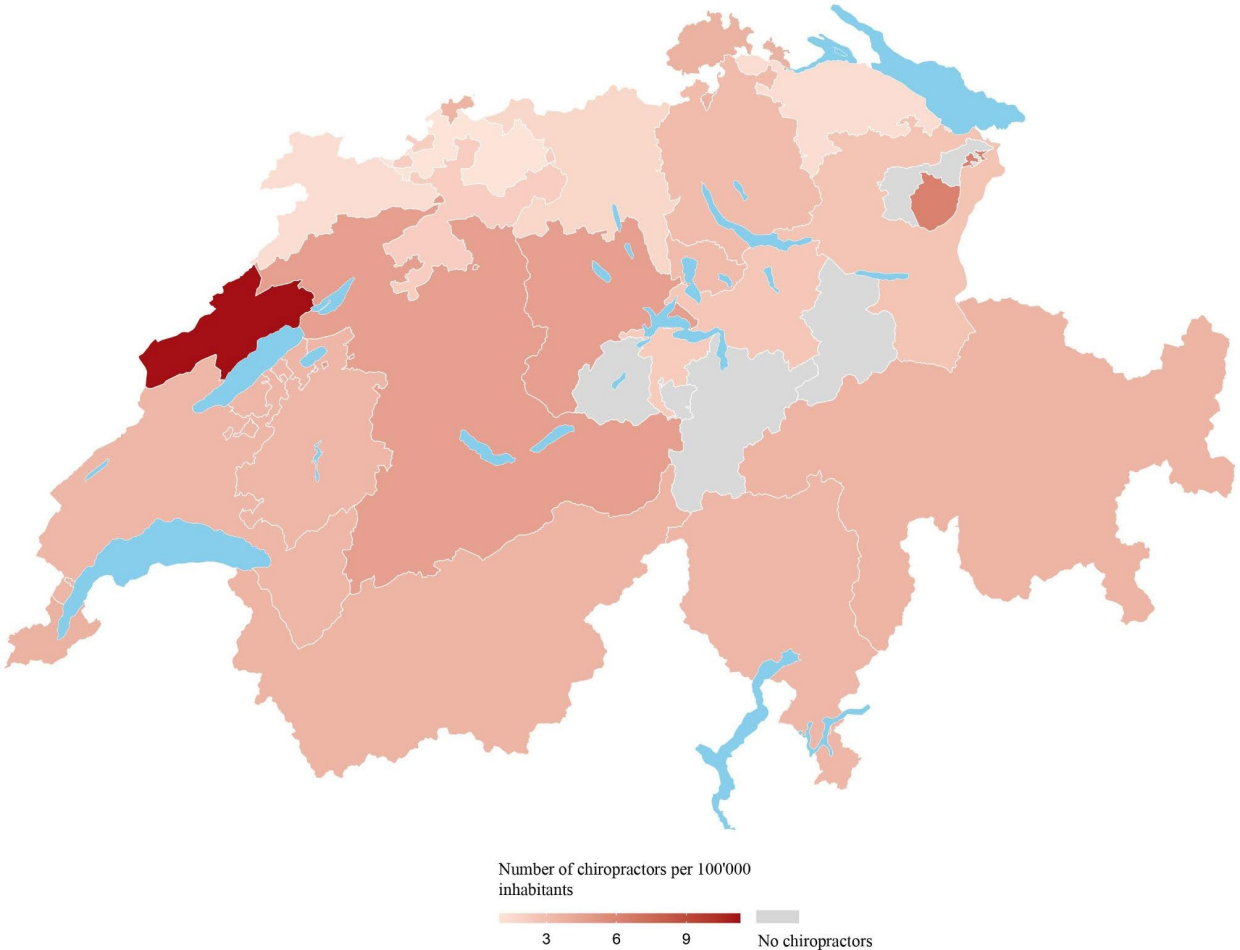
The geographic distribution of chiropractors in Switzerland, per 100,000 population in January 2020

The cantons with the most chiropractors in absolute terms were Bern and Zurich, with 48 chiropractors each, followed by Vaud with 27 chiropractors. These three cantons accounted for 43% (123/286) of all Swiss chiropractors from the source population. Four cantons—Appenzell Ausserrhoden, Glarus, Obwalden, and Uri—had no practicing chiropractors within their cantonal boundaries. The density of chiropractors was 3.3 per 100,000 inhabitants at the country level; ranged between 2.1 and 4.6 per 100,000 inhabitants across the 7 major (statistical) regions of Switzerland [16]; and ranged from 0 to a maximum of 11.3 per 100,000 inhabitants among Switzerland’s 26 cantons. Figure 1 presents the density of chiropractors per 100,000 population across Switzerland (see Supplementary Appendix eTable 7 for details).

## Discussion

Our cross-sectional study found that as of January 2020, less than 50% of respondent Swiss chiropractors used an EPR system in clinical practice, while 60% used encrypted email technology. Younger age and larger practices with more clinicians were factors associated with use of these healthcare digitalisation technologies. With respect to the geographic distribution of Swiss chiropractors, the density of chiropractors was estimated at 3.3 per 100,000 inhabitants in Switzerland and ranged from 2.1 to 4.6 per 100,000 inhabitants across the 7 major regions of Switzerland.

### Findings within context of existing evidence

In Switzerland, chiropractic is one of five university-based academic health professions, and focuses primarily on the musculoskeletal system [17]. Swiss chiropractors typically treat the same musculoskeletal pain conditions (e.g., back pain, neck pain) as their international colleagues using manual treatment techniques including spinal manipulation and mobilisation, education and advice, and soft tissue (i.e., myofascial) therapy [8, 18]. Key differences in clinical practice relative to other settings lie in the expanded scope of practice, increased referrals from medical doctors, and better integration within the healthcare system [7, 17, 18]. In the United States, use of EPRs in chiropractic practices was estimated by the American Chiropractic Association to be about 33% in 2010 [19]. Within the Swiss context, primary care physicians showed EPR adoption of roughly 23% in 2010 [4], and up to 45% in 2013 [20]. Smaller practices and older physicians were found to use EPR systems less often [4].

According to the International Organization for Standardization [21], an EPR can be defined as a repository of patient data in digital form, stored and exchanged securely, aiming to support efficient and quality integrated healthcare [22]. By supporting the systematic collection and storage of patient and health-related data, the EPR may help improve communication for clinical decision making in healthcare [23], reduce errors by automated alerts and prompts [24], and contribute to the development of harmonised health data that could be used to improve clinical practice and research [25]. The aggregation of nationwide electronic healthcare data may also facilitate population-level healthcare quality monitoring [11, 26]. A national report highlighted the needs for better availability of information and standardised quality and safety indicators of the Swiss healthcare system [27].

The use of EPR systems may enable the systematic collection of real-world routine clinical practice information and support the integration of quality measures using the gathered information [26]. However, some of the reported barriers hindering progress include clinician skepticism of the potential value of EPR systems, complexity and limitations of existing EPR systems, privacy and security concerns, time burden of EPR use, and high costs [6]. We found similar perceived barriers for EPR and encrypted email use among Swiss chiropractors (qualitative comments collected via our survey), with some raising concerns about (1) EPRs deteriorating the clinician-patient therapeutic alliance and relationship, (2) data privacy and security, and (3) an uncompelling cost-benefit value proposition. This was quite similar to the main barriers for information technology use reported among Swiss ambulatory care physicians in 2008 by Rosemann and colleagues [4]. Key recommendations for improving and promoting the Swiss eHealth system include fostering the development of an explicit health data strategy to help create the “right” mindset and attitudes, improving the healthcare information technologies infrastructure, harmonising data access procedures, clarifying privacy and consent issues, and aligning incentives with aims and goals [28].

Regarding the geographic distribution of Swiss chiropractors, to our knowledge, this is the first study to describe the distribution of the chiropractic healthcare workforce in Switzerland. A recently published international study about the chiropractic workforce reported the highest density of chiropractors in the US, estimated at 24 chiropractors per 100,000 inhabitants [29]. Other European countries were reported to have similar geographic distributions of chiropractors as in Switzerland [29]. For comparison, the density of general practitioners in Switzerland has been estimated at 106 per 100,000 inhabitants [30]. This descriptive secondary finding helps to inform the level of musculoskeletal healthcare service capacity in Switzerland with respect to chiropractors—an important health services datapoint given the integration with other Swiss medical professions and high burden from musculoskeletal pain conditions in Switzerland [7, 31].

### Relevance and implications

Since EPRs form the basis of any eHealth system, a comprehensive implementation of a well-structured, interoperable, and meaningfully useful EPR system is crucial for achieving health information exchange and healthcare practice supported by electronic processes [32]. This is reflected by the Swiss national eHealth strategy, which aims to encourage and promote digitalization within the health system [33].

A new law as of 1 January 2022, which requires newly licensed outpatient healthcare providers to join an EPR community, is yet another top-down policy nudge to encourage healthcare digitalization adoption in Swiss primary care, with more experienced outpatient service providers being exempt for now [5]. Most Swiss chiropractors have not been required to switch to an EPR system due to this new law—our survey reveals an experienced workforce with a mean 22 years (SD, 10 years) of clinical experience. As new chiropractors are licensed and enter the healthcare workforce over time, the prevalence of digitalization among Swiss chiropractors will increase, not only because of this new law requiring EPR use among newly licensed clinicians, but also because EPR and encrypted email use among Swiss chiropractors are associated with younger age, as our study found.

It may be helpful for clinicians looking to adopt digital healthcare technologies to select products with consideration of the Swiss eHealth strategy [33]. Currently, many products used by Swiss chiropractors have no integration to an interoperable national EPR system and no patient access portal features, which may hinder the full promise of a digitalized Swiss primary care community. To increase the use of EPRs and digital health technologies both in Switzerland and within other primary care health systems around the world [34, 35], several approaches could be helpful. First, the benefit of EPRs in daily routine care must be increased—for example, by decision support systems, reminder systems to enable proactive treatment of patients with chronic diseases, and tools to support healthcare quality improvement and monitoring of patient reported outcome and experience measures. In addition, adequate approaches to offer fair and reasonable reimbursement for practice-level time and financial investments related to digitalization have to be considered, such as additional payment for electronically generated, evidence-based quality indicators.

### Strengths and limitations

A key strength of our study was the high participation rate of 76% of the full population of Swiss chiropractors, suggesting excellent representativeness of our findings. Notable limitations are that we collected data via an electronic survey, which may have led to selective participation of chiropractors more comfortable with technology. We also limited our data collection to a brief and focused survey. This pragmatic decision was made to maximize clinician participation, although it limits the breadth of our findings.

Due to the limitations of our collected data, we cannot be certain why German-speaking clinicians were more likely to use encrypted email compared with French- or Italian-speaking clinicians (86% versus 14%). However, one explanation may be a greater uptake of the Swiss German-based HIN encrypted email technology among German-speaking chiropractors (94% market share). Encrypted email by HIN—a solution for sending sensitive data by email in compliance with Swiss data protection regulations—was founded in 1996, in Steinhausen, Canton Zug, and has developed its services primarily within the German-speaking region of Switzerland. Only as recently as 2020, did HIN establish a branch in Yverdon-les-Bains, Canton Vaud, to better serve the needs of French-speaking Switzerland (la Romandie) [36].

Our cross-sectional survey was conducted between December 2019 and January 2020, so we do not believe our findings to be seriously affected by the COVID-19 pandemic. The first positive case of COVID-19 in Switzerland was confirmed on 25 February 2020, and a State of Extraordinary Situation under the Federal Law of Epidemics was declared on 16 March 2020. Our study was conducted before the COVID-19 pandemic. Nonetheless, a more recent survey of 152 Swiss chiropractors conducted through the Swiss chiropractic practice-based research network (PBRN) between September 2021 and December 2021 [37], suggested increased adoption of EPR and encrypted email technologies among Swiss chiropractors, with 56% (95%CI, 48–64%) of participants reporting use of an EPR system, and 85% (95%CI, 78–90%) use of encrypted email communication. These findings, although promising, should be cautiously interpreted as the participation proportion in the 2021 PBRN survey [37] was lower than in the currently reported 2019 EPR survey (47% versus 76%), and may represent a different study population.

## Conclusions

As of January 2020, 286 chiropractors were available to provide musculoskeletal healthcare service across Switzerland. EPR and encrypted email health technologies were still relatively underused, with less than 50% of Swiss chiropractors reporting use of an EPR system and 60% encrypted email use in clinical practice. Better implementation of electronic health information technologies across the Swiss chiropractic community is possible and encouraged for musculoskeletal healthcare quality improvement and monitoring.

### Electronic supplementary material

Below is the link to the electronic supplementary material.

Supplementary Material 1

## Data Availability

The datasets used and/or analysed during the current study are available from the corresponding author on reasonable request.

## Abbreviations

CI: Confidence interval
EPR: Electronic patient record
HIN: Health Info Net
HRA: Human Research Act
IQR: Interquartile range
OR: Odds ratio
REDCap: Research electronic data capture
SD: Standard deviation
STROBE: Strengthening the reporting of observational studies in epidemiology
