# Correction: Use of electronic patient records and encrypted email patient communication among Swiss chiropractors: a population-based cross-sectional study

**DOI:** 10.1186/s12998-023-00513-0

**Published:** 2023-10-10

**Authors:** Cesar A. Hincapié, Léonie Hofstetter, Rahim Lalji, Longin Korner, Mireille C. Schläppi, Serafin Leemann

**Affiliations:** 1 EBPI-UWZH Musculoskeletal Epidemiology Research, Balgrist University Hospital and University of Zurich, Zurich, Zurich, Switzerland; 2 Epidemiology, Biostatistics and Prevention Institute (EBPI) University of Zurich, Zurich, Switzerland; 3 University Spine Centre Zurich (UWZH), Balgrist University Hospital and University of Zurich, Zurich, Switzerland; 4 Swiss Chiropractic Association (ChiroSuisse), Bern, Switzerland; 5 Epidemiology, Biostatistics and Prevention Institute (EBPI) and University Spine Centre Zurich (UWZH), University of Zurich and Balgrist University Hospital, Forchstrasse 340, Zurich 8008, Switzerland

**Chiropractic & Manual Therapies (2023) 31:21**

10.1186/s12998-023-00495-z

After publication of this article [1], the authors reported that the legend for Fig. 1 should read ‘Fig. 1 The geographic distribution of chiropractors in Switzerland, per 100,000 population in January 2020’ instead of ‘Fig. 1 Placeholder’.

The original article [1] has been corrected.

## References

[1] Hincapié CA, Hofstetter L, Lalji R, et al. Use of electronic patient records and encrypted email patient communication among Swiss chiropractors: a population-based cross-sectional study. Chiropr Man Therap 2023;31:21. https://doi.org/10.1186/s12998-023-00495-z. 10.1186/s12998-023-00495-z PMC10353203 37461087

