# Supplementary material for: Use of electronic patient records and encrypted email patient communication among Swiss chiropractors: a population-based cross-sectional study
Source: Chiropr Man Therap. 2023 Jul 17;31:21. doi: 10.1186/s12998-023-00495-z (PMC10353203; doi:10.1186/s12998-023-00495-z)
Supplement: Supplementary file 1 — Supplementary Material 1 [file 12998_2023_495_MOESM1_ESM.docx]

**Online Supplementary Appendix**

**Title:** Use of electronic patient records and encrypted email patient communication among Swiss chiropractors: a population-based cross-sectional study

**Authors:** Cesar A Hincapié DC PhD,^1,2,3^ Léonie Hofstetter DCM,^1^ Rahim Lalji DC MSc,^1,2,3^ Longin Korner DC,^4^ Mireille C Schläppi DC,^4^ Serafin Leemann DC^4^

**Affiliations:**

^1^ EBPI-UWZH Musculoskeletal Epidemiology Research Group, University of Zurich and Balgrist University Hospital, Zurich, Switzerland

^2^ Epidemiology, Biostatistics and Prevention Institute (EBPI), University of Zurich, Zurich, Switzerland

^3^ University Spine Centre Zurich (UWZH), Balgrist University Hospital, University of Zurich, Zurich, Switzerland

^4^ Swiss Chiropractic Association (ChiroSuisse), Bern, Switzerland

**Contents**

**eTable 1.** EPR products and manufacturers used by Swiss chiropractors as of 31.01.2020 2

**eTable 2.** Characteristics of top 5 EPR products used by Swiss chiropractors as of 31.01.2020 3

**eTable 3.** Billing products and manufacturers used by Swiss chiropractors as of 31.01.2020 4

**eTable 4.** Multiple logistic regression analysis of characteristics of Swiss chiropractors associated with EPR use 5

**eTable 5.** Characteristics of Swiss chiropractor participants, encrypted email users, and encrypted email non-users as of 31.01.2020 6

**eTable 6.** Multiple logistic regression analysis of characteristics of Swiss chiropractors associated with encrypted email use (N=217) 7

**eTable 7.** Geographic distribution of Swiss chiropractors (n=284) as of 31.01.2020 by country, region, and canton* 8

**Supplementary Appendix eTables**

# eTable 1. EPR products and manufacturers used by Swiss chiropractors as of 31.01.2020

| **Product** | **Manufacturer** | **N** | **%** |
| --- | --- | --- | --- |
| Pex | Delemed | 19 | 18.6 |
| siMed | Ametiq medical | 15 | 14.7 |
| MediOnline | AKCM | 13 | 12.7 |
| Vitomed | Vitodata AG | 12 | 11.8 |
| Chirwin | SwissChiroPool | 8 | 7.8 |
| Aeskulap | Kern Concept AG | 4 | 3.9 |
| mFchiro Evolution | Variosoft AG | 4 | 3.9 |
| Achilles | Axonlab AG | 3 | 2.9 |
| Mediway | Logival informatique | 3 | 2.9 |
| Praxistar | Praxinova Medizinal Informatik | 3 | 2.9 |
| Rockethealth | Helmedica AG | 3 | 2.9 |
| File maker | Claris International Inc. | 2 | 2.0 |
| Handy Patients | Handylife AG | 2 | 2.0 |
| MEDICOwin | InfoCall Produkt AG | 2 | 2.0 |
| Medidata | Medidata AG | 2 | 2.0 |
| PowerOffice | PowerOffice | 2 | 2.0 |
| Abacus | Abacus Research AG | 1 | 1.0 |
| KISIM | CISTEC AG | 1 | 1.0 |
| Medicaldesktop | MedicalDesktop AG | 1 | 1.0 |
| TriaMED | Swisscom Health AG | 1 | 1.0 |
| NA | NA | 1 | 1.0 |

# eTable 2. Characteristics of top 5 EPR products used by Swiss chiropractors as of 31.01.2020

|  | **Pex**  **(Delemed)** | **siMed**  **(Amétiq medical)** | **MediOnline**  **(AKCM)** | **Vitomed**  **(Vitodata AG)** | **Chirwin**  **(SwissChiroPool)** |
| --- | --- | --- | --- | --- | --- |
| Online appointment scheduling | Yes | Yes | Yes | Yes | Yes |
| Interfaces with digital x-ray and laboratory^1^ | Yes | Yes | Yes | Yes | Only x-ray |
| Integrated alert systems^2^ | Yes | Yes | Yes | Yes | No |
| Billing/invoicing | Yes | Yes | Yes | Yes | Yes |
| Statistics/reporting^3^ | Yes | Yes | Yes | Yes | Yes |
| Integration to interoperable EPR^4^ | Planned | Prepared | Yes | Yes | No |
| Patient access/portal^5^ | No | No | No | No | No |
| Connection of imaging modalities via integrated picture archiving and communication system. Integration of the laboratory order portal and automatic assignment of reports.  ^2^ Medication interaction check, clinical decision support system  ^3^ Business intelligence reporting, evaluation of administrative, medical, treatment-relevant and economic data  ^4^ Compliance, data transmission and connection to interoperable electronic patient record compatible with national standards  ^5^ Direct integration of a patient portal or application for patient data access and communication | | | | | |

# eTable 3. Billing products and manufacturers used by Swiss chiropractors as of 31.01.2020

| **Product** | **Manufacturer** | **N** | **%** |
| --- | --- | --- | --- |
| MediOnline/MediWin | AKCM | 41 | 18.9 |
| Medidata | Medidata AG | 37 | 17.1 |
| Pex | Delemed AG | 27 | 12.4 |
| Chirwin | SwissChiroPool | 22 | 10.1 |
| mFchiro Evolution | Variosoft AG | 4 | 3.9 |
| siMed | Ametiq medical | 14 | 6.5 |
| Achilles | Axonlab AG | 6 | 2.8 |
| PowerOffice | PowerOffice | 6 | 2.8 |
| Vitomed | Vitodata AG | 6 | 2.8 |
| Aeskulap | Kern Concept AG | 4 | 1.8 |
| Praxistar | Praxinova Medizinal Informatik | 2 | 0.9 |
| TriaMED/CuraMED | Swisscom Health AG | 2 | 0.9 |
| eChiropraktor | Tectis | 2 | 0.9 |
| Abacus | Abacus Research AG | 1 | 0.5 |
| Mediact | BBsoft | 1 | 0.5 |
| Confidis | Confidis | 1 | 0.5 |
| MEDICOwin | InfoCall Produkt AG | 1 | 0.5 |
| Mediway | Logival informatique | 1 | 0.5 |
| Outlook | Microsoft | 1 | 0.5 |
| OPALE | OPALE Solutions AG | 1 | 0.5 |
| Sumex | Sumex TIS TARMED | 1 | 0.5 |
| NA | NA | 20 | 9.2 |

eTable 4. Multiple logistic regression analysis of characteristics of Swiss chiropractors associated with EPR use (N=217)

| **Variable** | **OR** | **(95% CI)** |
| --- | --- | --- |
| Sex |  |  |
| Male | Referent |  |
| Female | 1.50 | 0.80 to 2.83 |
| Age group |  |  |
| ≤39 years | Referent |  |
| 40 to 59 years | 0.63 | 0.25 to 1.55 |
| ≥60 years | 0.26 | 0.08 to 0.77 |
| Language |  |  |
| German | Referent |  |
| French or Italian | 0.69 | 0.23 to 1.98 |
| Region |  |  |
| Zurich (CH04) | Referent |  |
| Lake Geneva region (CH01) | 1.48 | 0.34 to 6.56 |
| Espace Mittelland (CH02) | 0.94 | 0.35 to 2.55 |
| Northwestern Switzerland (CH03) | 0.60 | 0.15 to 2.24 |
| Eastern Switzerland (CH05) | 1.55 | 0.47 to 5.21 |
| Central Switzerland (CH06) | 0.60 | 0.17 to 1.99 |
| Ticino (CH07) | 2.21 | 0.35 to 14.99 |
| Number of chiropractors in clinic |  |  |
| 1 | Referent |  |
| 2 to 3 | 1.0 | 0.52 to 1.91 |
| ≥4 | 5.59 | 2.09 to 16.50 |

# eTable 5. Characteristics of Swiss chiropractor participants, encrypted email users, and encrypted email non-users as of 31.01.2020

| **Characteristic** | **Study population  (n=217)** | | **Encrypted email users (n=131)** | | **Encrypted email non-users (n=86)** | |
| --- | --- | --- | --- | --- | --- | --- |
|  | N | % | N | % | N | % |
| Sex | | | | | | |
| Female | 77 | 35.5 | 44 | 33.6 | 33 | 38.4 |
| Male | 140 | 64.5 | 87 | 66.4 | 53 | 61.6 |
| Age – mean ± SD (y) | 50.7 ± 11.2 | | 49.2 ± 9.7 | | 52.9 ± 12.8 | |
| Age (y) | | | | | | |
| ≤ 29 | 4 | 1.8 | 2 | 1.5 | 2 | 2.3 |
| 30 – 39 | 27 | 12.4 | 15 | 11.5 | 12 | 14.0 |
| 40 – 49 | 68 | 31.3 | 49 | 37.4 | 19 | 22.1 |
| 50 – 59 | 71 | 32.7 | 49 | 37.4 | 22 | 25.6 |
| 60 – 69 | 35 | 16.1 | 12 | 9.2 | 23 | 26.7 |
| ≥ 70 | 10 | 4.6 | 3 | 0.8 | 7 | 8.1 |
| NA | 2 | 0.9 | 1 | 0.8 | 1 | 1.2 |
| Language | | | | | | |
| German | 140 | 64.5 | 113 | 86.3 | 27 | 31.4 |
| French | 67 | 30.9 | 18 | 13.7 | 49 | 57.0 |
| Italian | 10 | 4.6 | 0 | 0 | 10 | 11.6 |
| Clinical experience – mean ± SD (y) | 22.1 ± 10.1 | | 21.0 ± 8.7 | | 23.9 ± 11.9 | |
| Clinical experience (y) | | | | | | |
| ≤10 | 28 | 12.9 | 15 | 11.5 | 13 | 15.1 |
| 11 – 20 | 71 | 32.7 | 52 | 39.7 | 19 | 22.1 |
| 21 – 30 | 79 | 36.4 | 48 | 36.6 | 31 | 36.0 |
| 31 – 40 | 28 | 12.9 | 14 | 10.7 | 14 | 16.3 |
| 41 – 50 | 9 | 4.1 | 1 | 0.8 | 8 | 9.3 |
| ≥ 50 | 1 | 0.5 | 0 | 0 | 1 | 1.2 |
| NA | 1 | 0.5 | 1 | 0.8 | 0 | 0 |
| Practice size – N chiropractors | | | | | | |
| 1 | 102 | 47.0 | 51 | 38.9 | 51 | 59.3 |
| 2 – 3 | 81 | 37.3 | 50 | 38.2 | 31 | 36.0 |
| ≥ 4 | 34 | 15.7 | 30 | 22.9 | 4 | 4.7 |

# eTable 6. Multiple logistic regression analysis of characteristics of Swiss chiropractors associated with encrypted email use (N=217)

| **Variable** | **OR** | **(95% CI)** |
| --- | --- | --- |
| Sex |  |  |
| Male | Referent |  |
| Female | 0.56 | 0.26 to 1.19 |
| Age group |  |  |
| ≤39 years | Referent |  |
| 40 to 59 years | 1.83 | 0.64 to 5.30 |
| ≥60 years | 0.25 | 0.07 to 0.86 |
| Language |  |  |
| German | Referent |  |
| French or Italian | 0.07 | 0.03 to 0.15 |
| Number of chiropractors in clinic |  |  |
| 1 | Referent |  |
| 2 to 3 | 1.03 | 0.47 to 2.22 |
| ≥4 | 7.36 | 2.04 to 33.0 |

# eTable 7. Geographic distribution of Swiss chiropractors (n=284) as of 31.01.2020 by country, region, and canton*

| **Geographic level** | **NUTS Code** | **Population** | **N chiropractors** | **Density per 100,000** |
| --- | --- | --- | --- | --- |
| Switzerland (NUTS-1) | CH0 | 8,606,033 | 284 | 3.3 |
|  |  |  |  |  |
| Region (NUTS-2) |  |  |  |  |
| Lake Geneva region | CH01 | 1,654,751 | 57 | 3.4 |
| Espace Mittelland | CH02 | 1,886,584 | 86 | 4.6 |
| Northwestern Switzerland | CH03 | 1,171,157 | 24 | 2.1 |
| Zurich | CH04 | 1,539,275 | 48 | 3.1 |
| Eastern Switzerland | CH05 | 1,183,813 | 29 | 2.5 |
| Central Switzerland | CH06 | 818,962 | 28 | 3.4 |
| Ticino | CH07 | 351,491 | 12 | 3.4 |
|  |  |  |  |  |
| Canton (NUTS-3) |  |  |  |  |
| Vaud | CH011 | 805,098 | 27 | 3.4 |
| Valais | CH012 | 345,525 | 12 | 3.5 |
| Geneva | CH013 | 504,128 | 18 | 3.6 |
| Berne | CH021 | 1,039,474 | 48 | 4.6 |
| Fribourg | CH022 | 321,783 | 11 | 3.4 |
| Solothurn | CH023 | 275,247 | 6 | 2.2 |
| Neuchâtel | CH024 | 176,496 | 20 | 11.3 |
| Jura | CH025 | 73,584 | 1 | 1.4 |
| Basel-Stadt | CH031 | 195,844 | 10 | 5.1 |
| Basel-Landschaft | CH032 | 289,468 | 2 | 0.7 |
| Aargau | CH033 | 685,845 | 12 | 1.8 |
| Zurich | CH040 | 1,539,275 | 48 | 3.1 |
| Glarus | CH051 | 40,590 | 0 | 0 |
| Schaffhausen | CH052 | 82,348 | 3 | 3.6 |
| Appenzell Ausserrhoden | CH053 | 55,445 | 0 | 0 |
| Appenzell Innerrhoden | CH054 | 16,128 | 1 | 6.2 |
| St. Gallen | CH055 | 510,734 | 14 | 2.7 |
| Grisons | CH056 | 199,021 | 7 | 3.5 |
| Thurgau | CH057 | 279,547 | 4 | 1.4 |
| Lucerne | CH061 | 413,120 | 19 | 4.6 |
| Uri | CH062 | 36,703 | 0 | 0 |
| Schwyz | CH063 | 160,480 | 4 | 2.5 |
| Obwalden | CH064 | 37,930 | 0 | 0 |
| Nidwalden | CH065 | 43,087 | 1 | 2.3 |
| Zug | CH066 | 127,642 | 4 | 3.1 |
| Ticino | CH070 | 351,491 | 12 | 3.4 |
| Abbreviations: *N*, number; *NUTS*, Nomenclature of Territorial Units for Statistics * Two chiropractors from Lichtenstein excluded | | | | |
